# Identification and Impact Analysis of Family History of Psychiatric Disorder in Mood Disorder Patients With Pretrained Language Model

**DOI:** 10.3389/fpsyt.2022.861930

**Published:** 2022-05-20

**Authors:** Cheng Wan, Xuewen Ge, Junjie Wang, Xin Zhang, Yun Yu, Jie Hu, Yun Liu, Hui Ma

**Affiliations:** ^1^Department of Medical Informatics, School of Biomedical Engineering and Informatics, Nanjing Medical University, Nanjing, China; ^2^Institute of Medical Informatics and Management, Nanjing Medical University, Nanjing, China; ^3^Department of Information, First Affiliated Hospital, Nanjing Medical University, Nanjing, China; ^4^Department of Medical Psychology, Affiliated Nanjing Brain Hospital, Nanjing Medical University, Nanjing, China

**Keywords:** family history, mood disorder, psychiatric disorder, electronic health records, pretrained BERT CNN model

## Abstract

Mood disorders are ubiquitous mental disorders with familial aggregation. Extracting family history of psychiatric disorders from large electronic hospitalization records is helpful for further study of onset characteristics among patients with a mood disorder. This study uses an observational clinical data set of in-patients of Nanjing Brain Hospital, affiliated with Nanjing Medical University, from the past 10 years. This paper proposes a pretrained language model: Bidirectional Encoder Representations from Transformers (BERT)–Convolutional Neural Network (CNN). We first project the electronic hospitalization records into a low-dimensional dense matrix *via* the pretrained Chinese BERT model, then feed the dense matrix into the stacked CNN layer to capture high-level features of texts; finally, we use the fully connected layer to extract family history based on high-level features. The accuracy of our BERT–CNN model was 97.12 ± 0.37% in the real-world data set from Nanjing Brain Hospital. We further studied the correlation between mood disorders and family history of psychiatric disorder.

## 1. Introduction

Mood disorders have high incidence and affect the health of people in China and worldwide (1). The main types of mood disorder are major depressive disorder (MDD) and bipolar disorder (BD) in depression; these have a great impact on individuals and families. In China, the lifetime prevalence of MDD is 6.9% and the 12-month prevalence is 3.6% (2). The lifetime suicide risk for MDD is 2.2–15% (3). The World Health Organization has estimated a lifetime prevalence of 1.0% for BD in 11 countries across the Americas, Europe, and Asia (4). Clinically, patients with MDD and BD in depression have similar manifestations, and diagnosis is complicated. Patients with BD spend approximately half of their lives symptomatic and the majority of that time suffering from symptoms of depression (5), which makes distinguishing BD from depression difficult. More convenient diagnostic tools are required to improve differential diagnosis of mood disorders.

Family history of psychiatric disorder is considered to be a major risk factor for most psychiatric disorders (6). Studies have reported heritability estimates of around 40% for MDD (7, 8) and 80% for BD (9, 10), indicating that these are highly hereditary conditions. In particular, the offspring of parents with MDD have a two- to five-fold increased risk of experiencing an episode of MDD (11, 12) and an increased risk of earlier onset of MDD (12).

With the continuous development of big data, disease-related family histories can be extracted from electronic health records (EHRs) to provide important data sources for clinical research (13, 14). A variety of family health history tools using quantitative survey data have been developed for use in clinical settings (15–17). However, family history is mostly documented in unstructured EHR narratives instead of structured fields.

Owing to the successful development of various deep learning (DL) techniques, accurate and effective DL-based natural language processing (NLP) systems can be built to alleviate this problem (18–22). On most NLP tasks, bidirectional transformer encoder representation (BERT) can achieve state-of-the-art performance while requiring minimal architectural modification (23). BERT and its derivatives, including Bert-wwm-text (24) and BioBERT (25), have achieved state-of-the-art results on various NLP tasks (e.g., question-answering, named entity recognition, and relation extraction) through simple fine-tuning techniques (26–28). However, few studies have explored the use of such models for extracting family history from EHRs, especially in Chinese language.

In this study, a clinical event classification model based on BERT with a convolutional neural network (CNN) (briefly BERT–CNN) is proposed to directly identify psychiatric disorder in the family history of patients in a manner convenient for observation and application. Data were collected from an observational clinical data set of in-patients at Nanjing Brain Hospital from the past 10 years. The distribution of psychiatric disorder in the family history of patients with severe depression is discussed, and the correlation between severe depression and family history of psychiatric disorder is studied.

## 2. Method

### 2.1. Data Set

#### 2.1.1. Data Source

**Figure 1:**
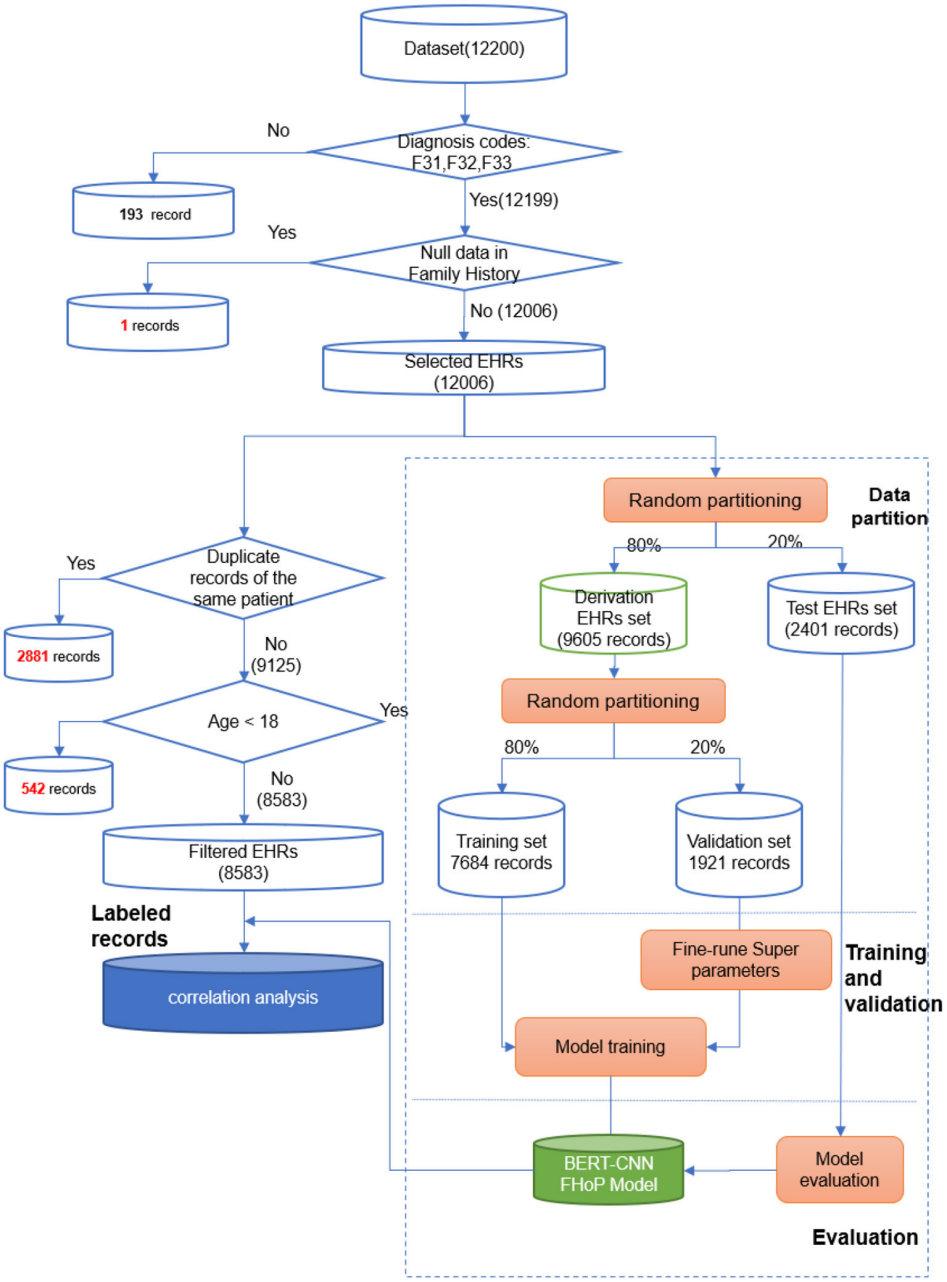
The research pipeline.

This study used the anonymous medical records of in-patients who visited the Nanjing Brain Hospital, affiliated with Nanjing Medical University, during the period from November 2010 to December 2019. The anonymous clinical notes were de-identified clinical notes where patients' private information was removed. The hospital is the largest psychiatric medical center in Jiangsu province, serving more than 8 million people in China. This time frame was chosen because the EHR system of the hospital achieved 99% completeness from 2010 onward. Patients' admission notes were extracted from the EHR system if their first diagnosis included “depression” in Chinese. The admission notes included patient's age, gender, marital status, profession, first diagnosis at admission, and a paragraph describing not only the patient's family history but also their present illness, past history, and results of personal physical examination. Admission notes were restricted to those of patients with diagnoses of MDD, recurrent MDD, or BD in depression. The International Classification of Diseases version 10 (ICD-10) codes of these three disease are F32, F33, and F31, respectively. The pipeline of the research is shown in Figure 1.

#### 2.1.2. Labeling Family History of Psychiatric Disorder

According to the disease name mentioned in the family history, admission notes were grouped into five subgroups: schizophrenia (F20), depressive episode (F32), BD (F31), other non-organic mental disorders (F28), and unspecified non-organic psychosis (F29). The label of each note was a quintet. For example, if a note included the sentence “the patient's sister had been diagnosed with a major depressive disorder,” it was labeled as (F20 = 0, F32 = 1, F31 = 0, F28 = 0, F29 = 0). Here, a note was annotated as positive if it described any diagnosis associated with family history of psychiatric disorder. Otherwise, it was annotated as negative. The definitions of kinship levels are given in Supplementary Table 1.

Labeling an admission included three steps. First, Jieba (29) was used for text segmentation of the admission notes. Jieba is one of the most popular Chinese text-segmentation tools, and the dictionary-based method using Jieba has been reported to achieve a precision of approximately 0.50–0.677 (30). Second, a rule-based algorithm was used to highlight family histories of psychiatric events in admission notes and output preliminary labels. Third, manually checking was used to verify labels and output the final labels as the gold standard for model evaluation and correlation analysis. Two psychiatrists reviewed the labels independently.

#### 2.1.3. Merging Family History of Psychiatric Disorder

For any patient with multiple hospitalization records, we identified all family history events from all the patients' admission notes and merged them as one family history label. For example, if there were three family history records for one patient, with schizophrenia mentioned in the first two records and MDD mentioned in the last, the patient's family history was labeled as (F20 = 1, F32 = 1, F31= 0, F28 = 0, F29 = 0). According to the first diagnosis name, patients' admission notes were grouped into three groups: BD in depression, MDD, or recurrent MDD. As there were fewer admission notes containing second and third kinship levels of family history, the first, second, and third degrees of kinship were merged into one group for the correlation analysis.

### 2.2. BERT–CNN Model for Identifying Family History

Qualifying admission notes containing descriptions of family history were randomly divided into a training set (64%), validation set (16%), and test set (20%) to build and evaluate the BERT–CNN model. All analyses of the model were implemented in Python (version 3.8.5). All Python code we developed for modeling is available upon request.

#### 2.2.1. Model Architecture

**Figure 2:**
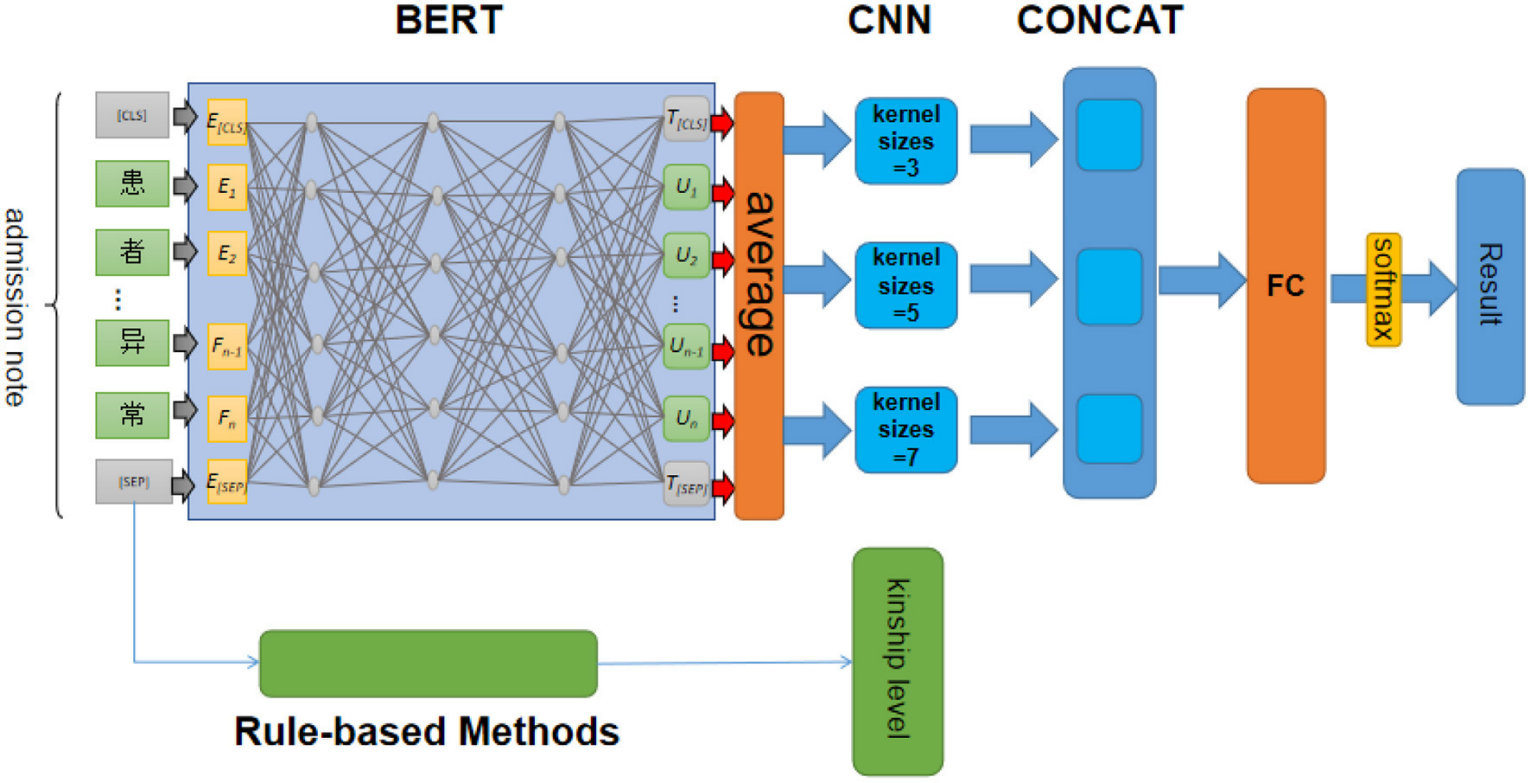
Architecture of the BERT–CNN model.

To extract family history of psychiatric disorder from hospitalization records effectively, we combined the pre-trained BERT model with a CNN network. The architecture of the model is shown in Figure 2. Rule-based methods were applied to extract the kinship level of family history. Disease names in family histories were extracted by the following steps. First, the Chinese pre-training language model BERT-wwm-ext released by the Harbin Institute of Technology IFLYTEK Joint Laboratory (24) was used to obtain a preliminary text semantic representation as a low-density dimensional matrix. Second, the obtained semantic representation vector for the family history text was used as an input to a CNN network, and convolution kernels of different dimensions were used to perform multi-dimensional feature extraction on the text, with ReLU activation and a stride of one. Third, max pooling was used to select the maximum value of the obtained feature vectors; then, the obtained feature vectors were put into the same dimension and combined. Finally, a full connection layer and sigmoid function were employed to identify the events of family history of psychiatric disorder mentioned in the sentence of interest. The output of the model is a quintet tuple obtained by a bit-wise OR operation on family history classification results across all admission notes for each patient.

#### 2.2.2. Parameter Settings

Super-parameters of the BERT–CNN model are described in Supplementary Table 2. For fine-tuning, except for batch size, learning rate, and the number of training periods, the model hyper-parameters were the same as those used in the pre-training model (31). In this study, a parameter grid search was used to identify the best-performing model; we set the number of training epochs to 100 and adjusted the other hyper-parameters to optimize the performance of the model. The hyper-parameter spaces were learning rate ∈{1*e*^−4^, 1*e*^−5^} and batch size ∈{16, 32}.

#### 2.2.3. Evaluation Metrics

The performance of the BERT–CNN model with different hyper-parameter combinations was evaluated with respect to accuracy, precision, recall, and F_1_. The definitions of these metrics are given in Supplementary Table 3. The macro average and micro average of precision, recall, and F_1_ were used because of the data imbalance. Here, macro average is simply the mean value of the different classes, whereas the micro version considers the class proportions as weights. The value ranges of all the metrics were from 0 to 1. Each reported performance metric was the average score of five runs with the same data splits randomly. Moreover, a receiver operating characteristic (ROC) curve was constructed for each model. We compared the performance of our model with two simpler models. One was a word2vec-CNN model that concatenated a word-to-vector embedding (word2vec) (32) and CNN; the other was a BERT–FC model that concatenated a pretrained BERT model and a fully connected (FC) layer.

### 2.3. Correlation Analysis

The associations between family history of psychiatric disorder and patient's first diagnosis at admission were analyzed using Pearson's chi-squared test at a 0.05 significance level. According to the etiology and clinical symptoms of the disease, both depressive episodes and recurrent depressive disorder were considered to belong to the category of depressive disorder (MDD). Therefore, all admission notes were classified into one of two groups, MDD (F32 and F33) or BD (F31), according to the code of the first diagnosis name. Multivariable step-wise binary logistic regression analysis was then performed to explore the potential correlations *via* which family history influenced the diagnosis of mood disorder. The outcome was a binary variable representing the first diagnosis at admission (MDD = 1 vs. BD = 0). The first diagnosis name was the first diagnosis recorded in the patients' admission notes. Commonly, the first diagnosis is the most important one. In the regression analysis, age, gender, marital status, and profession were used as potential risk factors. Here, age was the patients' age at admission; gender was male or female as recorded; marital status was self-reported as married, unmarried, bereft of a spouse, or other; and profession was also self-reported as one of 19 classes according to the name of the profession in Chinese. Odds ratios (OR) and 95% confidence intervals (95% CI) were calculated to assess the model's goodness of fit. All correlation analyses were implemented in R (version 3.6.2). All R code is available upon request.

## 3. Results

### 3.1. Participants

There were 12,200 anonymous clinical notes collected for this study, of which 194 admission notes were excluded from the analysis because of missing family history text or because they did not satisfy the criteria of this research. The average length of text in these admission notes was 204.3 (standard deviation [sd] 65.66) characters, with a maximum length of 948 characters and minimum length of 60 characters. The remaining 12,006 admission notes, which were used to create and evaluate the BERT–CNN model, included data for 7,850 female and 4,156 male patients ranging in age from 9 to 94 years, with an average age of 48.75 years. Admission time ranged from 2010 to 2019. Overall, there were 8694 patients with spouses, 3,296 patients without spouses, and 16 patients with no record of marital status. Various professions were mentioned in records; however, the largest group of patients consisted of retirees (approximately 26.2%). Regarding disease diagnosis, there were 2,123 cases of BD (F31), 5,353 cases of depression (F32), and 4,530 cases of recurrent MDD (F33) (see Table 1 for details).

**Table 1:** Demographic characteristics of the study participants.

	**BD in depression**	**Depression**	**Recurrent MDD**
Diagnosis names and ICD-10 codes of records	(F31)	(F32)	(F33)
*N*	2,123	5,353	4,530
Sex, *n* (%)			
Female	1,263 (16.0)	3,383 (43.0)	3,204 (41.0)
Male	860 (20.6)	1,970 (47.4)	1,326 (32.0)
Age (years), *n* (%)			
≤ 18	148 (18.7)	539 (68.4)	101 (12.9)
18–40	949 (30.6)	1,468 (47.2)	691 (22.2)
41–60	707 (16.2)	1,977 (45.6)	1,666 (38.2)
61–80	303 (8.6)	1,315 (36.9)	1,937 (54.5)
>80	16 (7.8)	54 (26.3)	135 (65.9)
Marital status, *n* (%)			
Married	1,179 (13.6)	3,855 (44.3)	3,660 (42.1)
No spouse	940 (28.6)	1,491 (45.2)	865 (26.2)
Not specified	4 (25.0)	7 (43.7)	5 (31.3)
**Events of family history of psychiatric disorder**			
–Disease name, *n* (%)			
Schizophrenia	145 (31.5)	151 (32.8)	169 (36.7)
Unspecified non-organic psychosis	26 (21.4)	58 (47.9)	37 (30.6)
MDD	177 (17.2)	419 (40.5)	437 (42.3)
BD	35 (59.4)	12 (20.3)	12 (20.3)
Other non-organic mental disorders	212 (32.4)	250 (38.2)	192 (29.4)
–Kinship level, *n* (%)			
First degree	368 (23.1)	593 (37.2)	632 (39.6)
Second degree	115 (33.6)	145 (42.3)	82 (23.9)
Third degree	18 (28.5)	23 (36.5)	22 (34.9)
Not mentioned	1,622 (16.2)	4,592 (45.8)	3,794 (37.9)

### 3.2. Performance of BERT–CNN in Identifying Family History of Psychiatric Disorder

The BERT–CNN model was used to identify family history of psychiatric disorder as described in the admission notes of in-patients. The model achieved an average accuracy of 0·971 (sd 3.72e-3), which was considered to be sufficient. Table 2 shows performance measures of the model during internal cross-validation with four super-parameter combinations. Overall, the micro metrics of F_1_, precision, and recall had values about 0.1 higher than those of the macro metrics, because the macro metrics did not take label imbalances into account. The micro metrics for F_1_, precision, and recall were 0.719 (SD 2.40e-2), 0.570 (SD 3.12e-2), 0.976 (SD 2.30e-2), and the best performance was achieved with a learning rate of 1e-4 and batch size of 16. The values for accuracy were higher than those for F1 and precision, owing to the imbalance of the data source. There were significantly fewer family history events of unspecified non-organic psychosis (F29, *N* = 121) compared with other disorders. The performance of the model under different super-parameter combinations was considered to be stable, because there was no significant difference between the results according to *t*-tests (*p* > 0.05). ROC curves and associated values for the model are provided in Supplementary Figure 2. Comparisons of Bert-CNN, Word2vec-CNN, and Bert-FC are provided in Supplementary Table 4.

**Table 2:** Performance measures of the model.

		**Batch size = 16**	**Batch size = 32**	**p^***a***^**
	**Learning rate = 1e-4**			
	Accuracy (sd)	0.971 (2.60e-3)	0.970 (5.86e-3)	
	F_1_ (sd)	0.721 (2.03e-2)	0.708 (3055e-2)	
	Precision (sd)	0.568 (2.39e-2)	0.559 (5.13e-2)	
Micro	Recall (sd)	0.989 (1.41e-2)	0.973 (1.92e-2)	0.851
	F_1_(sd)	0.668 (5.26e-2)	0.606 (5.84e-2)	
	Precision(sd)	0.563 (9.03e-2)	0.515 (1.32e-1)	
Macro	Recall(sd)	0.936 (7.23e-3)	0.841 (6.87e-3)	0.296
	**Learning rate = 1e-5**			
	Accuracy (sd)	0.972 (1.98e-3)	0.972 (1.82e-3)	
	F_1_ (sd)	0.724 (1.41e-2)	0.722 (1.25e-2)	
	Precision (sd)	0.576 (1.32e-2)	0.576 (1.10e-2)	
Micro	Recall(sd)	0.973 (2.29e-2)	0.969 (2.64e-2)	0.973
	F_1_ (sd)	0.630 (1.32e-2)	0.613 (8.27e-2)	
	Precision (sd)	0.559 (1.32e-1)	0.524 (1.42e-1)	
Macro	Recall(sd)	0.847 (1.11e-2)	0.828 (1.27e-2)	0.701

a *p>0.05 indicates stable performance of the model under different super-parameter combinations*.

**Figure 3:**
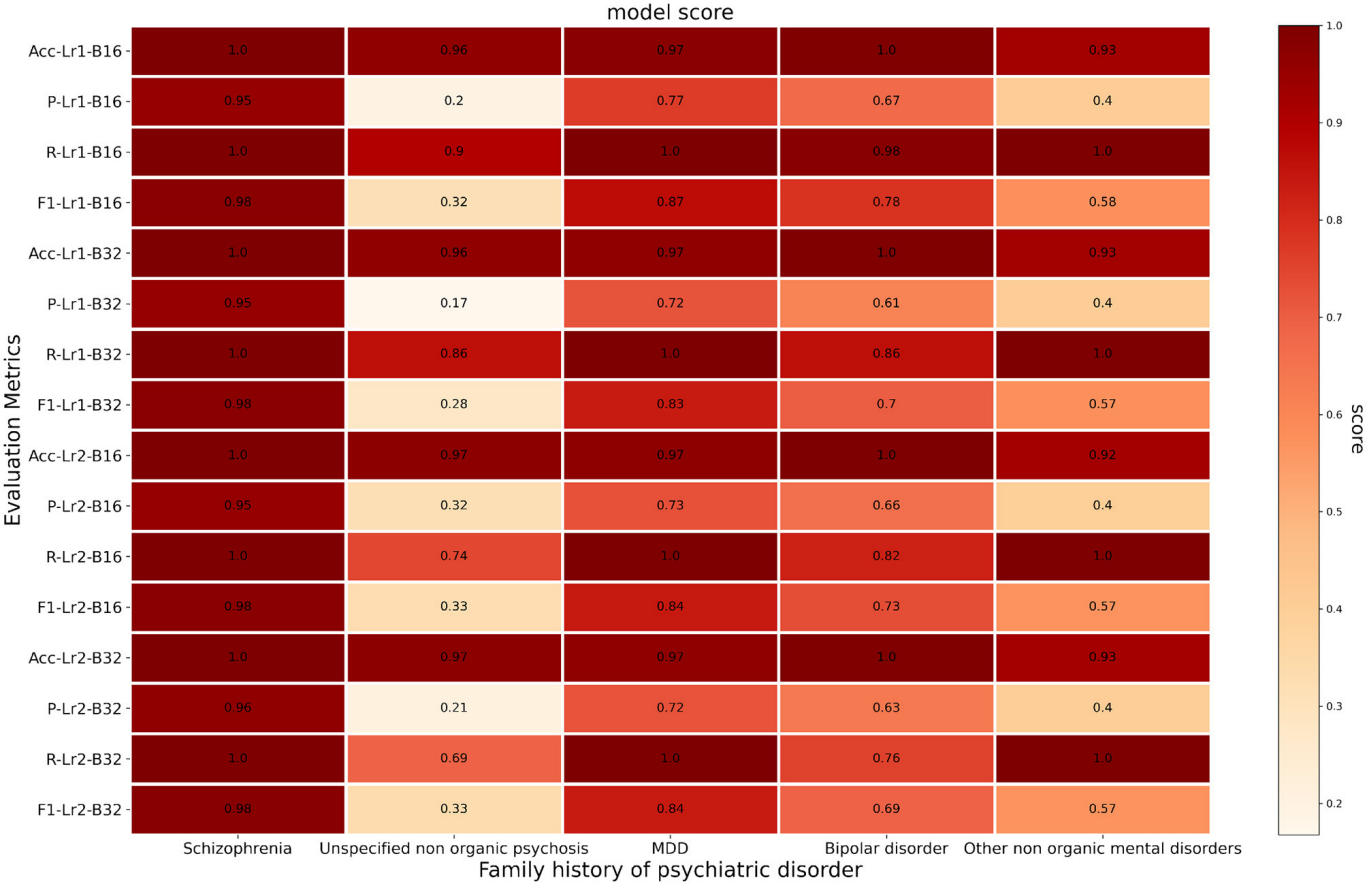
Heatmap representation of disorder extraction scores from different evaluation metrics. Acc, accuracy; P, precision; R, recall; Lr1, learning rate = 1e-4; Lr2, learning rate = 1e-5; B16, batch size = 16; B32, batch size = 32.

In general, our model performed well in correctly labeling different psychiatric disorders in the family histories of patients. Figure 3 shows the performance of the model in extracting different names of psychiatric disorders from family histories using the BERT–CNN model, including the accuracy, precision, recall, and F_1_ for each disorder in detail. As shown in the figure, the best performance was achieved in the identification of schizophrenia for each evaluation metric. The mean accuracy, precision, recall, and F_1_ values were 1, 0.95, 1, and 0.98, respectively. The model showed relatively modest precision, as it could not correctly identify one specific description, as shown in Supplementary Figure 1. The recall of the fine-tuned model with learning rate 1e-4 and batch size 16 was high enough for all the positive admission notes to satisfy the requirement of labeling psychiatric diseases in the patient's family history and to reduce the time required for manual screening.

In order to better visualize the attention patterns in each layer of the pretrained model, the bertviz (19) tool was adapted. We added “SEP” tokens between family history labels and the full admission notes only for visualization purposes. In the pretrained model, among the 12 layers of the BERT-www-mnx transformer model, in the middle three layers (4–6), some medically meaningful attention patterns that captured contextual and visit-dependent information emerged. An example of how codes are connected with each other according to the attention weights from the transformer layers is shown in Supplementary Figure 3.

### 3.3. Correlation Analysis

There were significant differences between family history of psychiatric disorder and first diagnosis (chi-squared contingency table test statistic: 293.98, *p* < 2.2e-16. After excluding the influence of gender, age, profession, and marital status, we found correlations of family history of BD, schizophrenia, and other non-organic mental disorders with patients' first diagnosis (Table 3). MDD as the first diagnosis at admission was significant negative correlation with family history of schizophrenia (OR = 0.464, 95% CI: 0.356–0.611), family history of BD (OR = 0.137, 95% CI: 0.071–0.264), and family history of other non-organic mental disorders (OR = 0.409, 95% CI: 0.332–0.506). Patients with a family history of schizophrenia were less likely to develop MDD than BD. However, MDD as the first diagnosis at admission was no significant correlation with family history of MDD (OR = 1.058, 95% CI: 0.851–1.327, *p* = 0.617) and family history of unspecified non-organic psychosis (OR = 0.643, 95% CI: 0.368–1.180, *p* = 0.135).

**Table 3:** Risk of MDD as first diagnosis at admission presented as adjusted ORs grouped by family history of psychiatric disorder.

	**OR^***a***^**	**95% CI**	***p*** **-value**
Family history of MDD			
Yes	1.058	0.851–1.327	0.617
No	1.00 (ref.)	1.00 (ref.)	
Family history of schizophrenia			
Yes	0.464	0.356–0.611	<0.05
No	1.00 (ref.)	1.00 (ref.)	
Family history of BD			
Yes	0.137	0.071–0.264	<0.05
No	1.00 (ref.)	1.00 (ref.)	
Family history of unspecified non-organic psychosis			
Yes	0.643	0.368–1.180	0.135
No	1.00 (ref.)	1.00 (ref.)	
Family history of other non-organic mental disorders			
Yes	0.409	0.332–0506	<0.05
No	1.00 (ref.)	1.00 (ref.)	

a *Adjusted for sex, age, marital status, and profession*.

## 4. Discussion

As the etiology of mood disorders is unclear, their diagnosis mainly depends on the clinical diagnosis classification systems. It is difficult to make an accurate diagnosis of BD in clinical practice because 75% of BD patients initially present with a depressive episode. Accordingly, the misdiagnosis rate of BD is very high (33). Our study found that a positive family history of schizophrenia, BD, and other non-organic mental disorders was positively associated with BD in depression as the patient's first diagnosis at admission. Genes have a moderate role in the etiology of mood disorders. Large empirical studies of the genetic architectures of MDD and BD indicate that they are polygenic. It has been suggested previously that patients with a family history of psychiatric diagnoses may have higher risk of BD (34).

However, family histories of psychiatric patients are mainly saved in text form in many hospitals' EHR systems. It is challenging to use the abundant information in EHRs owing to its high dimensionality, noise, heterogeneity, sparseness, incompleteness, and susceptibility to random errors and systematic biases (20). Recently, developments in the field of natural language processing, especially in methods based on DL models, have provided powerful tools to extract useful information automatically. In this study, the BERT–CNN model was used to directly extract and structure psychiatric disorder information from the family histories of patients. This approach will provide a convenient method for further clinical research.

The BERT–CNN model showed good performance in event-extraction-based raw text analysis of observational clinical records of mood disorder patients. Data labeling involves a huge workload when conducting observational research on routine secondary or tertiary care data. The simpler labeling method proposed in this paper could widen the application scope of the BERT–CNN model. First, event-based extraction instead of named entity recognition was used in the extraction of family history in order to reduce the workload required. Models based on named entity recognition have been tried but showed no significant difference in performance compared with those shown in Table 2. Second, raw text from the admission notes was used directly in this study. The text in the admission notes extracted from the hospital's EHR system included not only family history but also the patient's current and past medical history, results of personal physical examinations, etc. These texts are concatenated as one paragraph. However, there was no significant difference in the model's performance when only shorter sentences containing family history extracted from the whole paragraph were used, compared with the BERT–CNN model. Third, the BERT–CNN model achieved higher overall accuracy (acc = 0.971) compared with simpler models, e.g., the word2vec-CNN model (acc = 0.81) and BERT–FC model (acc = 0.610). Although the word2Vec-CNN model performed slightly higher on two metrics compared with BERT–CNN (0.011 higher on micro-F1 and 0.09 higher on micro-precision), the BERT–CNN model performed better on the remaining five metrics. It also performed better on all metrics compared with the BERT–FC model. The BERT pre-training model achieves better word vector expression than word2vec and then obtains better global information in sentences through the use of CNN, resulting in superior performance overall.

The accuracy of the BERT–CNN model was good enough (higher than 0.92 in every psychiatric disorder) to identify all the family histories of psychiatric disorders. Unfortunately, the precision of the model was not good enough in identifying unspecified non-organic psychosis and other non-organic mental disorders. This was because the model performed well only on specific types of description of family history. If a rule to identify this situation were added to the BERT–CNN model, the resulting hybrid BERT–CNN model would have an improved precision of 93%. The difficulty of the model in handling certain information may have been because the Chinese pre-training language model BERT-wwm-ext had not undergone enough training with clinical texts. The Alibaba Group has published another Chinese biomedical pre-training model, ChineseBLUE (35). The pre-training data used for this model were from Chinese medical Q&A, Chinese medical encyclopedias, and Chinese electronic medical records. Among the 20M sentences used in ChineseBLUE, only 10k sentences were from electronic medical records, which might not be enough for clinical applications. The intelligent medical research group of the artificial intelligence research center of Pengcheng Laboratory published PCL-MedBERT in 2020; they stated that 1.2G of professional medical texts, collected from multiple sources, were used to train this model. However, despite our best efforts, we could not find details of the clinical texts used as the source information for the model's pre-training. Accordingly, we suggest that more pre-training models for clinical text processing based on Chinese observational clinical data should be developed and made available.

Data imbalance is a typical feature of observational data that can dramatically influence the performance of DL models. The data set used for the BERT–CNN model had this feature. The micro-averaged metrics of our model thus produced better results than the macro versions because of the imbalance between classes: there were 465 cases of schizophrenia, 1033 cases of MDD, and 654 cases of other non-organic mental disorders, but only 121 cases of unspecified non-organic psychosis and 59 cases of BD. This suggests that better balancing algorithms should be developed to solve the unbalanced data problem that is frequently encountered in observational clinical text research.

Our study had some limitations. Our model did not extract full family histories but focused on family history of psychiatric disorder only. Further experiments will be performed to identify more disorders. Furthermore, the pre-trained BERT model used in this study was a Chinese pre-training language model that used Chinese Wikipedia (simplified and traditional) for training, rather than models trained by a clinical text data set. A pre-trained model based on a big data set of clinical texts will be developed in our future work. In addition, the data set was only from the Nanjing Brain Hospital affiliated with Nanjing Medical University. More data from other hospitals will be used to validate and improve the performance of the BERT–CNN model in future.

## 5. Conclusion

In this paper, a new model, BERT–CNN, was proposed to automatically extract psychiatric family history disorders from patient hospitalization records. Experimental results showed that the BERT–CNN model achieved good performance in the extraction of family history of psychiatric disorders. The family history information extracted by the model from observational electronic records from a 10-year period suggested that patients with a family history of psychiatry may have higher risk of BD.

## Data Availability Statement

The datasets presented in this article are not readily available because data privacy and security requirements. Requests to access the datasets should be directed to HM, mahui@njmu.edu.cn.

## Ethics Statement

The studies involving human participants were reviewed and approved by Medical Ethics Committee of Nanjing Brain Hospital (2020-KY147-01). Written informed consent from the [patients/participants OR patients/participants legal guardian/next of kin] was not required to participate in this study in accordance with the national legislation and the institutional requirements.

## Author Contributions

CW and HM designed the study. XG did the experiments of deep-learning models. CW and XG drafted the manuscript and did the statistical analysis. JW provided guidance on the BERT model. XZ, YY, and JH critically revised the manuscript for important intellectual content. YL and HM provided guidance on the significance of disease research. All authors acquired, analyzed, interpreted data, provided administrative, technical, and material support. All authors contributed to the article and approved the submitted version.

## Funding

This work was supported by the industry prospecting and common key technology key projects of Jiangsu Province Science and Technology Department (Grant no. BE2020721), the National Key Research & Development plan of Ministry of Science and Technology of China (Grant nos. 2018YFC1314900 and 2018YFC1314901), the big data industry development pilot demonstration project of Ministry of Industry and Information Technology of China [Grant nos. (2019)243 and (2020)84], the Industrial and Information Industry Transformation and Upgrading Guiding Fund of Jiangsu Economy and Information Technology Commission [Grant no. (2018)0419], and Jiangsu Province Engineering Research Center of Big Data Application in Chronic Disease and Intelligent Health Service [Grant no. (2020)1460]. The corresponding author had full access to all the data in the study. All authors had final responsibility for the decision to submit the manuscript for publication.

## Conflict of Interest

The authors declare that the research was conducted in the absence of any commercial or financial relationships that could be construed as a potential conflict of interest.

## Publisher's Note

All claims expressed in this article are solely those of the authors and do not necessarily represent those of their affiliated organizations, or those of the publisher, the editors and the reviewers. Any product that may be evaluated in this article, or claim that may be made by its manufacturer, is not guaranteed or endorsed by the publisher.

## Abbreviations

EHRs: Electronic hospitalization records
BERT: Bidirectional encoder representations from transformers
CNN: Convolutional neural network
MDD: Major depressive disorder
FC: Fully connected
BD: Bipolar disorder
DL: Deep learning
NLP: Natural language processing
ICD: International classification of diseases
Word2Vec: Word to vector
OR: Odds ratio
CI: Confidence interval.
