# Supplementary material for: Identification and Impact Analysis of Family History of Psychiatric Disorder in Mood Disorder Patients With Pretrained Language Model
Source: Front Psychiatry. 2022 May 20;13:861930. doi: 10.3389/fpsyt.2022.861930 (PMC9163373; doi:10.3389/fpsyt.2022.861930)
Supplement: Supplementary file 1 [file Data_Sheet_1.pdf]

Supplementary Material

1 SUPPLEMENTARY DATA

1.1 Tables

Table S1. Definitions of kinship levels

Kinship level	Definition
First-degree	Relatives sharing 50% of the patient's DNA:<br>parents, children, siblings
Second-degree	Relatives sharing 25% of the patient's DNA:<br>grandpa, grandma, uncle, aunt, grandfather, grandmother, grandchild
Third-degree	Relatives sharing 12.5% of the patient's DNA:<br>great-grandfather, great-grandmother, cousin

Table S2. Hyper-parameter settings in BERT-CNN

Hyper-parameter	Value
Learning rate	$\{1e^{-4}, 1e^{-5}\}$
Batch size	$\{16, 32\}$
attention_probs_dropout_prob	0.1
directionality	bidi
hidden_act	gelu
hidden_dropout_prob	0.1
hidden_size	768
initializer_range	0.02
intermediate_size	3072
max_position_embeddings	512
num_attention_heads	12
num_hidden_layers	12
pooler_fc_size	768
pooler_num_attention_heads	12
pooler_num_fc_layers	3
pooler_size_per_head	128
pooler_type	first_token_transform
type_vocab_size	2
vocab_size	21128

Table S3. Definition of metrics

Metrics	Definition
TP	The number of admission notes in which a positive family history of psychiatric disorders was dentified as the correct positive subgroup by the model
TN	The number of admission notes in which a negative family history of psychiatric disorders was identified as the correct negative subgroup by the model
FP	Number of admission notes in which a negative family history of psychiatric disorder was identified as positive and those with a positive family history of psychiatric disorder was identified as wrong subgroup
FN	Number of admission notes in which a positive family history of psychiatric disorder was identified as negative by the model
Accuracy	(TP+TN)/(TP+TN+FP+FN) describes the percentage of correctly identified records among all data
Precision	TP/(TP+FP) describes the fraction of the number of true positive records among all positive records identified by the model
micro precision	Calculate precision globally by counting the total true positives, and false positives.
macro precision	Calculate precision for each label, and find their unweighted mean.
Recall	TP/(TP+FN) describes the fraction of the number of true positive records among all positive records
micro recall	Calculate recall globally by counting the total true positives, and false negatives.
macro precision	Calculate recall for each label, and find their unweighted mean.
Specificity	TN/(FP+TN) describes the fraction of the number of true negative records among all negative records
F_1	2*Precision*Recall/(Precision+Recall); the harmonic mean of the recall and precision
micro F_1	Calculate F_1 globally by counting the total true positives, false negatives and falsepositives.
macro F_1	Calculate F_1 for each label, and find their unweighted mean.This does not take labelimbalance into account.

Table S4. Comparison of the effects of different models.

		Bert-CNN	Word2vec-CNN	Bert-FC
	accuracy	0.971	0.81	0.61
micro	precision	0.570	0.66	0.067
	recall	0.976	0.81	0.781
	f1	0.719	0.73	0.123
macro	precision	0.540	0.41	0.054
	recall	0.863	0.50	0.476
	f1	0.680	0.45	0.089

Table S5. Complete results of logistic regression analysis.

		OR	95% CI	p-value
Age		1.65	1.023-1.037	<0.05
Gender				
	Female	1.106	0.971-1.259	0.128
Marital status				
	Married	0.970	0.599-1.496	0.897
	Unmarried	0.473	0.278-0.772	<0.05
	Bereft of spouse	0.460	0.280-0.815	<0.05
	Other marital status	0.408	0.120-2.265	0.285
Family history				
	Insanity	1.030	0.331-0.505	<0.05
	Psychosis	0.644	0.368-1.182	0.137
	Schizophrenia	0.465	0.356-0.612	<0.05
	Bipolar disorder	0.137	0.070-0.264	<0.05
Profession				
	staff	1.203	0.757-1.860	0.420
	doctor	1.530	0.644-4.093	0.361
	worker	0.969	0.606-1.509	0.892
	student	1.521	0.934-2.417	0.083
	engineer	0.402	0.175-0.950	<0.05
	freelancer	1.777	0.541-8.071	0.389
	individual workers	0.867	0.475-1.588	0.643
	individual operator	2.055	0.972-4.674	0.070
	civil servant	1.475	0.779-2.856	0.239
	national civil servant	0.323	0.096-1.202	0.074
	retired	1.176	0.741-1.812	0.477
	unemployed	0.890	0.570-1.345	0.592
	retired personnel	0.610	0.227-1.945	0.358
	active duty military	1.461	0.403-6.954	0.590
	professional skill worker	1.597	0.481-7.290	0.486

1.2 Figures

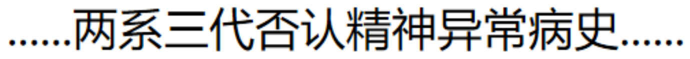

Figure S1: One special description

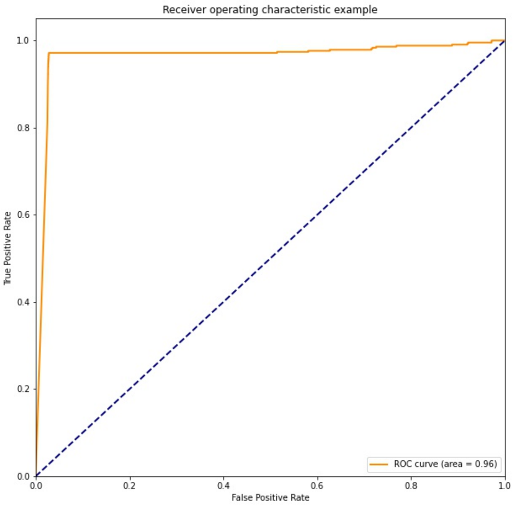

batch_size=32,learning_rate=$1e^{-4}$
AUC = 0.96

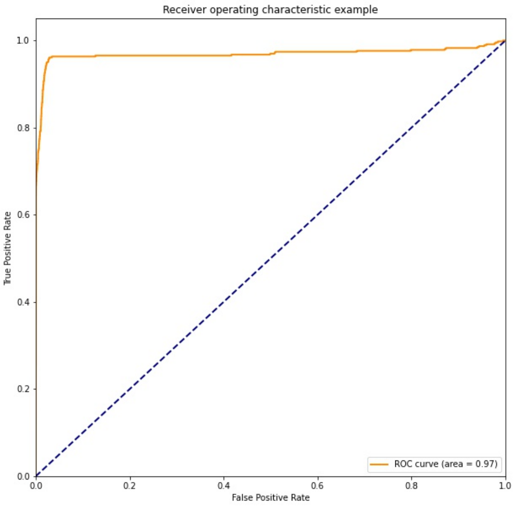

batch_size=16,learning_rate=$1e^{-4}$
AUC = 0.97

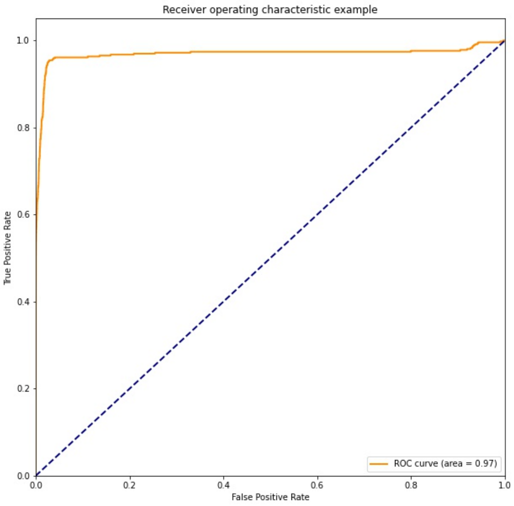

batch_size=32,learning_rate=$1e^{-5}$
AUC = 0.97

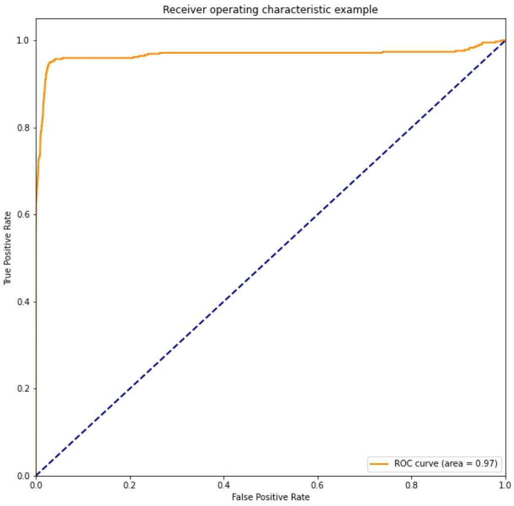

batch_size=16,learning_rate=$1e^{-5}$
AUC = 0.97

Figure S2: ROC curves of the models under different conditions

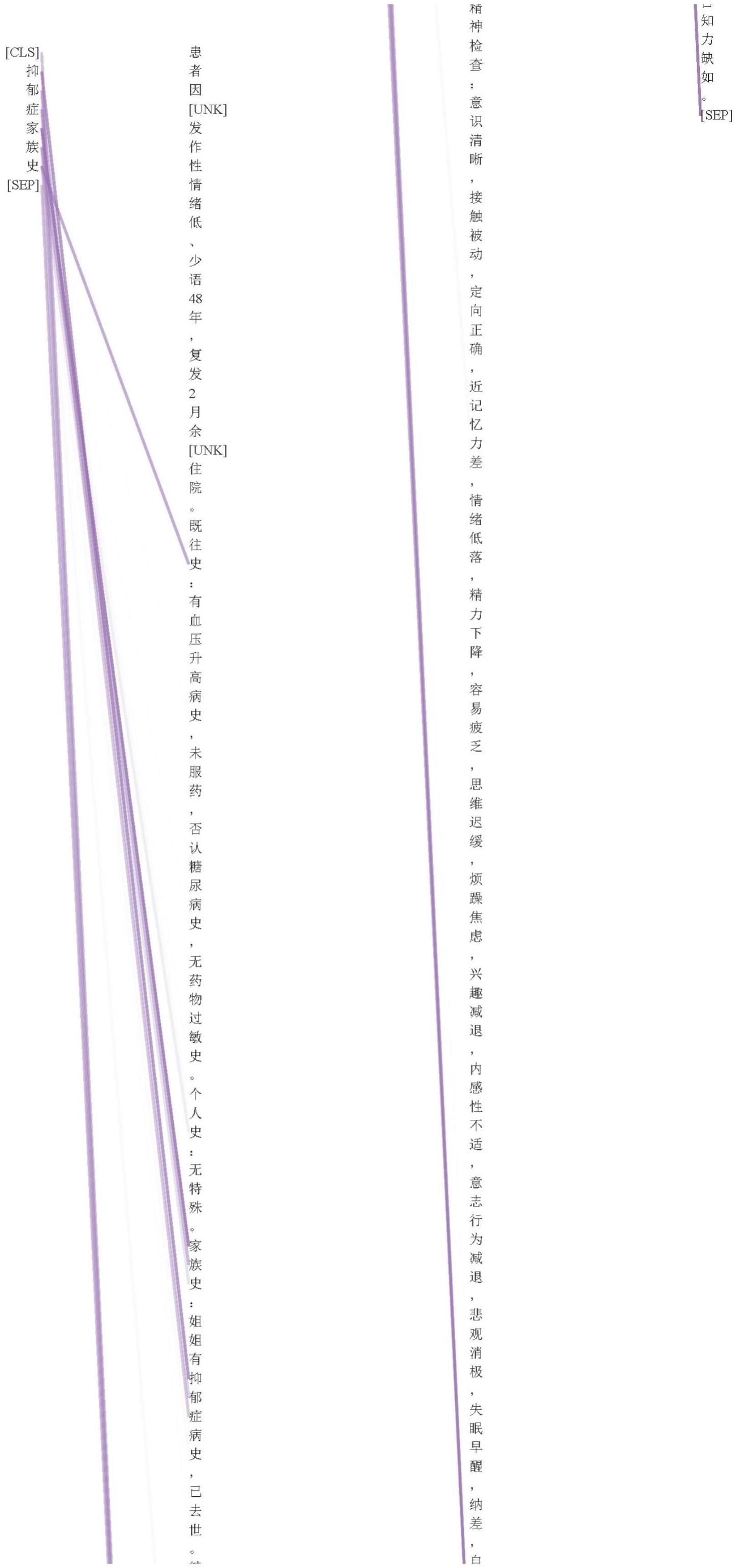

Figure S3: Visualization of the model
