# Supplementary figures and images for: Identification and Impact Analysis of Family History of Psychiatric Disorder in Mood Disorder Patients With Pretrained Language Model
Source: Front Psychiatry. 2022 May 20;13:861930. doi: 10.3389/fpsyt.2022.861930 (PMC9163373; doi:10.3389/fpsyt.2022.861930)

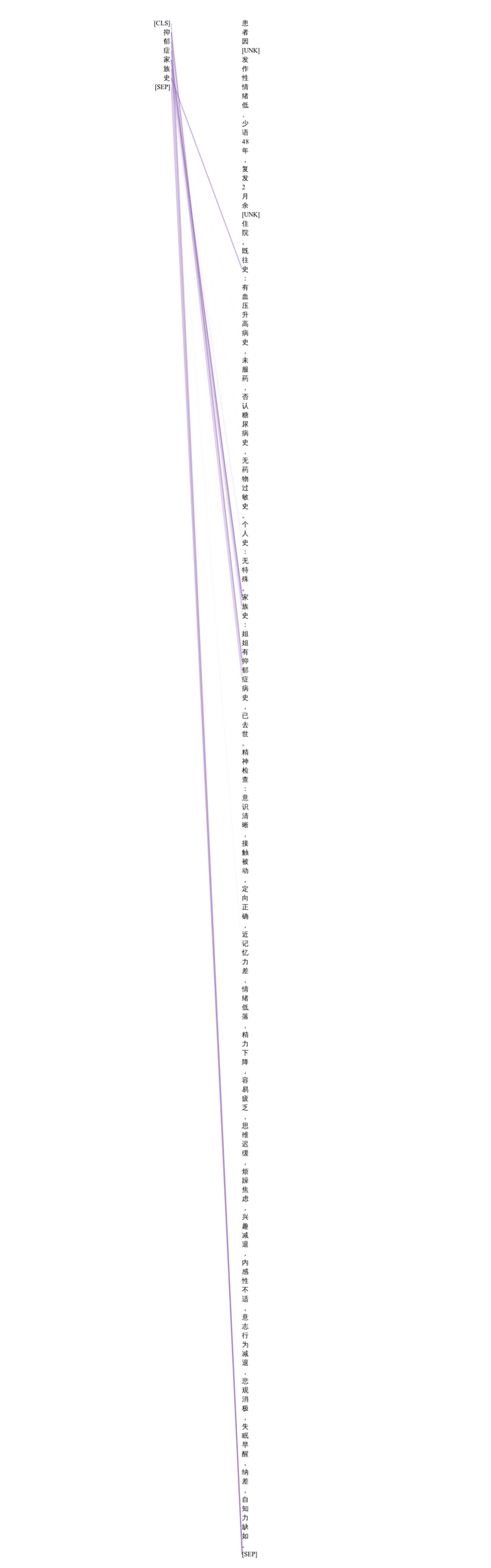

Supplement: Supplementary file 2 [file Image_1.PNG]
